# Engaging End Users to Inform the Design and Social Marketing Strategy for a Web-Based Sexually Transmitted Infection/Blood-Borne Virus (STI/BBV) Testing Service for Young People in Victoria, Australia: Qualitative Study

**DOI:** 10.2196/63822

**Published:** 2025-03-27

**Authors:** Ethan T Cardwell, Teralynn Ludwick, Shanton Chang, Olivia Walsh, Megan Lim, Rachel Podbury, David Evans, Christopher K Fairley, Fabian Y S Kong, Jane S Hocking

**Affiliations:** 1 Melbourne School of Population and Global Health Centre for Epidemiology and Biostatistics The University of Melbourne Parkville Australia; 2 School of Computing and Information Systems University of Melbourne Parkville Australia; 3 Burnet Institute Melbourne Australia; 4 School of Population Health and Preventive Medicine Monash University Melbourne Australia; 5 Paper Giant Melbourne Australia; 6 Matanuska-Susitna College University of Alaska System Anchorage United States; 7 Department of Rural Health Center For Excellence in Rural Sexual Health The University of Melbourne Shepparton Australia; 8 Alfred Health Melbourne Sexual Health Centre Melbourne Australia; 9 School of Translational Medicine Monash University Melbourne Australia

**Keywords:** web-based STI/HIV testing, social marketing, sexual health, participatory design, codesign, sexually transmitted infections, STI, HIV, Australia, social media, survey, blood-borne viruses

## Abstract

**Background:**

The rates of sexually transmitted infections (STIs) continue to rise across Australia among 16- to 29-year-olds. Timely testing is needed to reduce transmission, but sexual health clinics are at capacity. This demand, coupled with barriers to getting tested faced by young people, has led to web-based services as a pragmatic solution. However, for young people to use these services, they must be acceptable, attractive, and usable. Social marketing principles combined with end user engagement can be used to guide the development of a web-based service and create a marketing strategy to attract them to the service.

**Objective:**

Working closely with end users and guided by social marketing, this project explored messaging, design elements (imagery), and promotional strategies that will support high usage of a web-based STI/blood-borne virus (BBV) testing service for young people in Victoria, Australia.

**Methods:**

Young people were recruited to participate in half-day workshops via youth organizations and targeted Meta (Facebook/Instagram) advertisements. An initial web-based survey was deployed to inform workshop content. Workshops were held in metropolitan, outer metropolitan, and regional Victoria. Young people were presented with a range of “image territories” developed by a social marketing firm and social marketing messages that were informed by the literature on communicating health messages. Participants discussed the feelings and reactions evoked by the content. Data collected through mixed methods (transcribed notes, audio recording, and physical outputs) were thematically analyzed to understand features of messaging and imagery that would attract young people to use the service.

**Results:**

A total of 45 people completed the initial survey with 17 participating in focus group workshops (metropolitan: n=8, outer metropolitan: n=6, and regional: n=3). Young people preferred messages that highlight the functional benefits (confidential, affordable, and accessible) of a web-based service and include professional imagery and logos that elicit trust. Young people indicated that the service should be promoted through digital communications (eg, dating apps and social media), with endorsement from government or other recognized institutions, and via word-of-mouth communications.

**Conclusions:**

This study has highlighted the value of applying social marketing theory with end user engagement in developing a web-based STI/BBV testing service. Through the voices of young people, we have established the foundations to inform the design and marketing for Victoria’s first publicly funded web-based STI/BBV testing clinic. Future research will measure the reach and efficacy of social marketing, and how this service complements existing services in increasing STI/BBV testing uptake among young Victorians.

## Introduction

### Background

After a drop-off during the COVID-19 pandemic, sexually transmitted infections (STI) diagnosis rates are rising again throughout high-income countries and the burden of these infections is particularly high among young people aged 16-29 years [1]. The increased demand for STI and blood-borne virus (BBV) testing has placed a strain on sexual health services and led to the emergence of web-based services that are now increasingly common in the United States, United Kingdom, Canada, and Europe [2] as a solution for increasing access to testing.

While web-based services can be effective at increasing access to STI testing, they are not always acceptable or accessible to particular population groups, such as those living in regional or remote areas, and this is often because they have been developed with little engagement from the end user [3]. This lack of engagement with end users can explain usability problems, low uptake, and high attrition rates [3]—people simply stop using technologies that do not correspond with their daily lives or habits. Social marketing theory [4] and end user engagement [5] approaches are valuable for putting the focus on the end user, increasing the effectiveness, accessibility, and sustainability of web-based technologies [6]. Few studies have applied social marketing theory with end user engagement; however, researchers in the field have acknowledged the use of end user engagement and social marketing theory individually and the potential for synergy when used together [7,8].

### Development of a Web-Based STI/BBV Testing Service in Australia

To address the growing demand for access to STI/BBV testing in Australia, we are developing a fully automated web-based STI/BBV testing service to be offered as part of the only specialist sexual health service in Victoria, Australia, based in Melbourne (population of 5 million), the capital city of the State of Victoria (population of 6.7 million). Given the location of the specialist service in Melbourne, people living in regional Victoria have limited access to specialist STI/BBV care, placing them at considerable disadvantage. The aim of our web-based service is to increase STI/BBV testing uptake by providing access to specialist STI/BBV expertise anywhere across the State of Victoria, not just in Melbourne, particularly for young people aged 16-29 years. Our service will allow users to login to a website portal, answer risk-based consultation questions, receive a pathology request according to recommended testing guidelines [9], and have their results sent directly to them.

**Figure 1:**
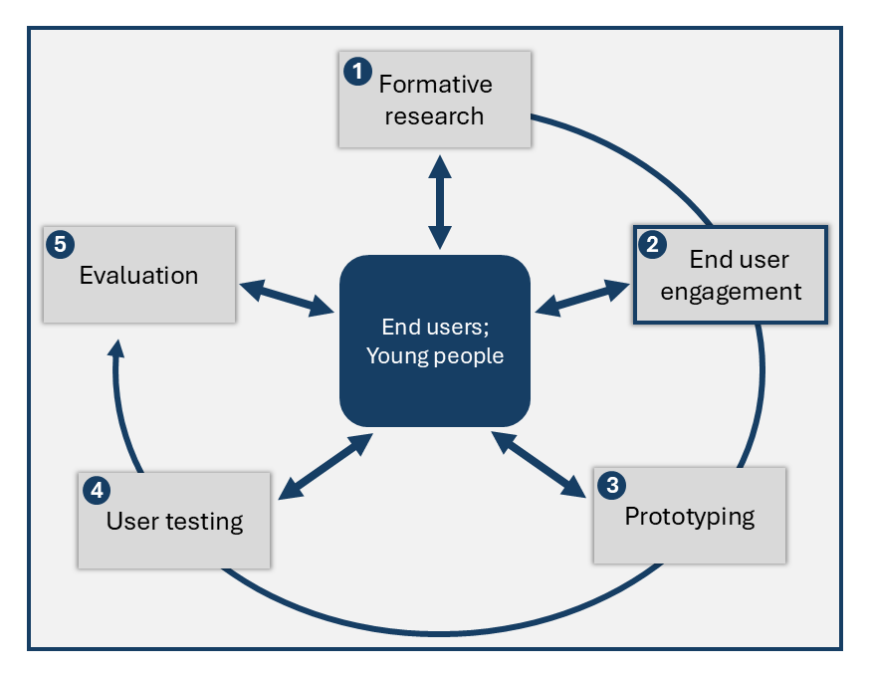
Five-phase approach to user-centered development of a web-based sexually transmitted infection/blood-borne virus testing service for young people aged 18-29 years in Victoria, Australia.

Development and implementation of our web-based service have five phases: (1) formative research in which we surveyed young people and reviewed existing web-based services in Australia to inform the design of our service [10-13]; (2) end user engagement in which we ran focus group workshops (workshops) to design the content and the tone of voice (language used), imagery, and branding of the service; (3) prototyping in which we are developing digital prototypes based on the findings from phases 1 and 2; (4) user testing in which we test the prototype with end users to assess its usability, acceptability, and refine development; and (5) evaluation in which we implement the web-based service and assess its impact on STI/BBV testing and diagnosis and its cost-effectiveness (Figure 1).

In this paper, we present the methods and results for phase 2, end user engagement, in which we had direct and iterative involvement with young people to explore the messaging and imagery design elements that would attract young people to use a web-based STI/BBV testing service and the best way to promote this messaging to young people. Further publications will detail our research in developing the user experience along the clinical pathway (part of phase 2), user testing of our web-based service (phase 4), and its subsequent evaluation (phase 5).

## Methods

### Study Design

In phase 2, we have used Andreasen’s theory of social marketing [4] to inform our approach to develop the imagery and messaging for our web-based STI/BBV service and have applied Andreasen’s six benchmarks as follows:

1. Behavior change: in our context, we want to ultimately increase STI/BBV test uptake among young people using the service; however, this will not be assessed until phase 5.
2. Formative research: we conducted surveys and interviews and reviewed available web-based services to understand young people’s experiences, values, and needs of a web-based service.
3. Segmentation: we targeted particular subgroups of young people to capture their views (viz, young people living in metropolitan and regional areas) based on our formative research [12], which showed differences in preferences between regional and metropolitan youth for how web-based STI/BBV services are delivered. By targeting these groups, we sought to understand how these differences would apply to the design and social marketing of our web-based service.
4. Marketing mix: we assess the service we are providing and how to price, place, and promote it.
5. Exchange: we assess what will motivate young people to engage with the service and what service they will get in return.
6. Competition: we assess competing behaviors and strategies to help us understand what would make a young person use our web-based testing service in preference to other testing services [4]. Our formative research showed that young people want to be able to access web-based STI/BBV testing services in addition to in-person services [12] because of limited availability of specialist in-person sexual health services and the time delays and cost often associated with accessing other in-person primary care services in Australia [2,10]. However, there are few web-based STI/BBV testing services in Victoria, and those that are available are rarely free, nor do they offer a comprehensive STI/BBV testing service in which all guideline-recommended tests are available [10].

Although our web-based service will be directed to people aged 16-29 years, people aged 18-29 years living in the State of Victoria were eligible for participation in phase 2 because of consent challenges when working with 16- and 17-year-olds in focus groups. Phase 2 involved three key steps:

1. An expression of interest (EOI) in which young people were invited to express their interest in being involved in the study. The EOI was advertised through youth service organizations across Victoria and targeted advertising on Meta (Facebook and Instagram). Those interested completed a brief web-based survey, which collected information about sociodemographics including their age, gender, sexuality, and residential postcode. The purpose of the EOI was to allow for purposive sampling for the focus group workshops, ensuring diverse representation of young people (eg, gender and sexuality) from across Victoria including metropolitan and regional locations. We reviewed EOIs in real time and placed additional advertisements targeting both young people living in regional areas and heterosexual men to attract more applicants from these demographic backgrounds as needed.
2. A self-administered web-based survey was completed by a select sample of respondents from the EOI. The demographic profile of EOI responses was screened as they were received, with approximately 15 individuals from each geographic location being invited to participate. The survey explored (1) social marketing including the preferred tone of voice, types of messaging, and image territories (group of images representing a design theme) that would attract young people to use a web-based STI/BBV testing service, and (2) the “user experience” along the clinical pathway from establishing a web-based account, answering sexual history questions, and receiving an STI/BBV form for testing. These survey responses were used to refine the messaging and image territories to explore in the focus group workshops. All individuals who completed the survey were invited to participate in the focus group workshops.
3. Focus group workshops, in which we further explored their preferred social marketing and user experience to inform the development of the web-based service with a sample of young people who completed the web-based survey. Workshop participants provided electronic or written consent and were reimbursed for their time. We conducted three 6-hour workshops that were segmented by geographical area with 1 in metropolitan area, 1 in outer metropolitan area, and 1 in regional areas. Metropolitan and outer metropolitan workshops were conducted on the university campus and the regional workshop was conducted at community organization in regional Victoria.

#### Testing Social Marketing Messaging

The web-based survey explored participants’ views about 8 social marketing messages that we developed informed from the literature on communicating health messages to young people [14-16]. Each message included aligns with one of the following approaches to messaging: provision of choice (provides choice between 2 or more behaviors), gain- or loss-framed (emphasizes the advantage or consequence of an action), value based (elicits a sensation of value), and other referencing (focuses on how an individual’s choice may affect others) [14-16]. A full list of messages can be found in Multimedia Appendix 1.

Each participant was asked to imagine themselves in 7 different scenarios and then pick the top 3 messages that would encourage them to seek a web-based STI/BBV testing service. Scenarios placed the user in an experience known from the literature as a facilitator or barrier to getting tested, such as stigma, self-efficacy, or risk perception [17-19]. These scenarios were based on the theory of planned behavior and the transtheoretical model [20]. A full list of the scenarios can be found in Multimedia Appendix 1.

The following four messages were the most selected:

- Message 1: Getting tested can protect your partners from STIs.
- Message 2: Confidential, affordable STI testing. Find out more!
- Message 3: Itching. Burning. Rash. Sores. Getting tested is a MUST!
- Message 4: Be healthy. Be safe. Get tested.

In the workshop, participants were presented with each message individually and asked their initial reactions to the message, how it may influence them to use the service, and how it may be improved. After all messages were presented, the participants voted on which of the 4 messages they liked the most.

#### Testing Image Territories

**Figure 2:**
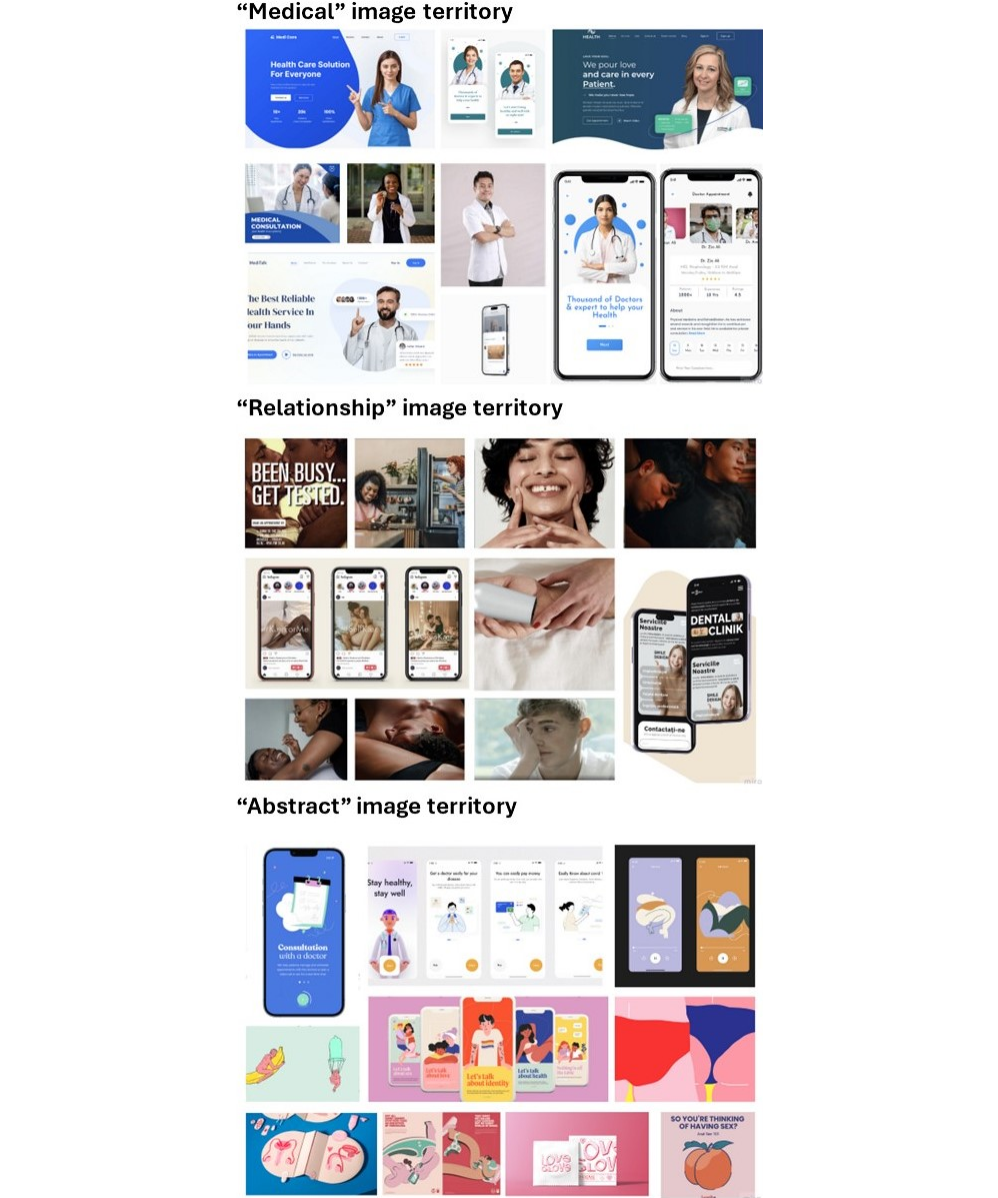
Three image territories used to explore design preferences during focus group workshops with young people aged 18-29 years in Victoria, Australia.

In our survey, we presented the participants with some examples of web-based service-landing pages (the first web page users see after clicking a link to a testing website) of 2 currently existing sexual health websites and asked them to choose which they liked best (Multimedia Appendix 1). They were then asked what they liked about the website they chose and how it could be improved. We then engaged a social marketing firm that developed 3 “image territories” (medical, relationship, and abstract) to test with young people (Figure 2). During our workshops, we explored how participants felt about each image territory and which one ultimately attracted them most to use this type of service.

#### Informing a Marketing or Promotion Strategy

To inform a strategy to attract young people to use the service, we explored (1) how participants find out about health services in their day-to-day life, (2) what factors influence their trust of these services, and (3) how they would like to find out about this service.

### Analysis

Data collected from the focus group workshops (transcribed notes, audio recording, and physical outputs) were thematically analyzed in Excel by ETC in consultation with SC using an inductive approach [21]. We validated high-level data with those attending the workshop (OW, RP, and TL). Illustrative quotes are presented by geographical location of the workshops. During the workshops the service was referred to as “online” or “web-based” interchangeably for simplicity.

### Ethical Considerations

All activities conducted in this study were approved by the Office of Research Ethics and Integrity at the University of Melbourne (2023-25277-43562-5). All participants were provided with plain language statements and signed an informed consent prior to participation in the study. The consent outlined the activities of the study and informed participants that all data collected would be deidentified and securely stored with password protection. Participants received Aus $20 (Aus $1.00=US $0.63) for completing the initial survey and an additional Aus $100 (Aus $1.00=US $0.63) electronic gift card for each 3-hour workshop they completed for a maximum remuneration of Aus $220 (Aus $1.00=US $0.63).

## Results

### Participants

A total of 45 individuals completed the initial survey and all were invited to attend a focus group workshop with 17 participating—8 in metropolitan, 6 in outer metropolitan, and 3 in regional locations. Age of workshop participants ranged from 18 to 29 years, with diverse gender (6 women, 9 men, 1 gender diverse, and 1 transgender), sexual orientations (6 heterosexual and 9 same-sex–attracted), and culturally and linguistically diverse backgrounds (7 culturally and linguistically diverse individuals) represented. Results from the focus group workshops are shown in the subsequent section.

### Social Marketing Messaging

Four themes emerged from analysis: functional benefits, tone, structure, and altruistic versus individual motivations. These themes were mentioned consistently across all workshops, and there was a general consensus among participants about the messaging they preferred. Participants had more in-depth discussion about the importance of functional benefits and tone than about the themes of structure and individual versus individual motivations.

#### Functional Benefits—Confidentiality, Affordability, and Accessible Web-Based STI Testing

Functional benefits refer to benefits that particularly relate to the performance of the service and subthemes of confidential, affordable, and specificity (online and STI testing), which were frequently mentioned as important to participants. Participants across all workshops made many comments about the importance of messaging having the functional benefits of the service. They felt that the addition of words “online” or “STIs” would be more descriptive of the service and keep messages from coming off as too “generic.”

[Be healthy. Be safe. Get tested.] I feel like context is important to make it obvious you're talking about STIs.

Metropolitan

It was clear that participants found it important to include aspects of the service that would be seen as valuable to users such as “confidential” and “affordable.” This was one of the key reasons for the last message, “Confidential, affordable STI testing. Find out more!” being their favorite.

[Confidential, affordable STI testing. Find out more!] Perfect, clear and concise, reiterates the service is confidential, affordable. Assures people can access, like university students.

Regional

Although we did not test a message that was specific to accessibility, participants indicated that the word “online” had implicit connotations of being easier to access in comparison with in-person services. In particular, the regional young people spoke about the difficulty of accessing timely health care in their area due to overstretched health care services.

Trying to find a doctor in a rural town is very hard because they’re not taking new clients.

Regional

#### Tone—Preference for a Nonjudgmental, Comforting Tone

Tone refers to the perception of how the messages come across to the reader with subthemes of commanding, passive, judgmental, comfort, and gimmicky identified. The tone used was brought up across all focus group workshops for every message. Participants did not like messages they perceived to be judgmental or gimmicky. Across all workshops the message, “Itching. Burning. Rash. Sores. Getting tested is a MUST!” was seen as judgmental and not a message they wanted to see. Some participants found certain phrases in the messages to be a bit judgy such as “Be healthy” or gimmicky such as “Find out more!”

[Itching. Burning. Rash. Sores. Getting tested is a MUST!] I don’t like it at all. If I already had itching and burning, I would already feel uncomfortable and awkward and wouldn't wanted to be shouted at.

Metropolitan

In addition, participants discussed the importance of striking a balance between a “passive” and “commanding” tone. The message should not come off as “yelling” or “commanding” but should not be so passive that it does not motivate young people to get tested using the service.

[Be healthy. Be safe. Get tested.] I think it's good that it's not fear mongering but maybe too passive.

Metropolitan

#### Structure—Preference for a Short and Snappy Message Structure With Inclusive Wording

Structure refers to the basic elements of the message with subthemes of length, grammar, and wording. The way the message was structured, such as length and grammar, was less important but played a role in how the message was perceived. Participant overall preferred when messages were “short and snappy” and felt that the messages should easily be consumed “if you’re walking past.” Punctuation was mostly important to participants because it affected the tone of the message. They found that the use of an exclamation and even a period in some places came off as too commanding or gimmicky.

[Itching. Burning. Rash. Sores. Getting tested is a MUST!] Exclamation is too much, feels like a WWII poster.

Metropolitan

Participants also highlighted the importance of word choice and how certain words such as “partners” may have a “different meaning to different people” and cause some confusion as if the service is right for them.

I know a lot of people would just look past it and say, I don’t have a partner, I've hooked up with 3 people in 3 days.

Regional

#### Altruistic Versus Individualistic Motivations

This theme refers to participants’ comments on the focus of the message being on the individuals themselves or altruistic in nature and how this affects their motivation to get tested. Most comments in this theme related to the message, “Getting tested can protect your partners from STIs.” There were mixed opinions from participants as to whether a message focused on the individuals themselves or focused on protecting another person would be more motivating for them.

It’s focused around other people so I think it would appeal to me more or influence me more to get tested.

Outer metropolitan

You’ll miss selfish people because [they would] possibly be interested in the service for selfish reasons.

Metropolitan

### Imagery—a Balance of Medical and Abstract Image Territories

Upon thematic analysis, 2 general themes of design neutrality and traditional and modern design emerged across all image territories. Neutral design refers to the generalizability of the design to young people from different backgrounds with young people preferring gender neutral color schemes. Traditional design refers to the professional medical advertisements that have been traditionally used for medical services, where modern refers to more youthful, abstract designs. Participants wanted to find a balance between the 2 as each had its own benefit.

I think it’s like any other doctor booking website service so if people respond to that then you can use the traditional methods. Why make it look like everything else when it’s targeting a niche[r] market.

Metropolitan referring to the Medical image territory

There were also themes for each of the image territories. The medical image territory used stock images of medical professionals and traditional depictions of the web-based clinical setting with blue and white color scheme with themes of trust and generic emerging. Although participants found these images to be generic “like any other medical website,” they did give participants a feeling of trust for the same reason, as they align with familiar medical service websites.

Very professional—too professional. It seems with the doctors and medical professionals makes it reliable and trustable.

Outer metropolitan referring to the Medical image territory

The relationship image territory used humanizing images of young people with some appearing to be in relationships and an earth tone color scheme. Participant found the color scheme to be too “luxe” and the images to be overly sexualized like it was “for a dating app.”

I won’t recommend this design at all. I just feel like it would be a dating and relationship website.

Outer metropolitan referring to the Relationship image territory

The abstract image territory used an animated approach to imaging with a pastel color scheme and themes of animation and comfort emerged. Participants found the animated images to bring them a sense of comfort as the images are more “relaxed” and relatable to other websites they use and like. However, they did find them feminine and geared toward “young women.”

Makes me feel more comfortable. I like the animated style, it doesn’t feel as daunting. The one thing I didn’t like—the colour combos make it feel more targeted to younger females.

Regional referring to the Abstract image territory

### Promoting and Marketing the Web-Based Service

When asked “How do you find out about health services in your day-to-day life?” participants mentioned social media, word of mouth from friends or health care providers, general searches on Google or specified health engines, and collateral materials posted at various physical locations. Using a search engine and through social media, including advertisements, were mentioned most by young people.

I’ve got ads that were given to me for health services that I wasn’t following. I think it’s helpful because you’re only getting them for a reason.

Metropolitan

We then asked participants how they would like to find out about this service particularly. Participants said similar methods to finding out about health services in general but focused more on digital advertising such as social media and dating apps.

Dating apps or porn websites; you’re going to where your audience is.

Metropolitan

We explored what influences their trust in these services. Participants commented that endorsement from friends, partnerships with existing professional organizations, and web-based reviews are important influences for trusting health services, particularly those on the web. It was clear that if participants had a certain level of trust with who they hear about the service from or who it is partnered with they were more likely to trust the service.

Word of mouth; people only go to practitioners they’ve had a good experience with.

Metropolitan

If it was endorsed by the government or some big hospitals then I would trust.

Outer metropolitan

## Discussion

### Principal Findings

Combining social marketing theory with end user engagement, we were able to identify key messaging and imagery that would attract young people to use a web-based STI/BBV testing service. We found that young people preferred messages that highlight the functional benefits (confidential, affordable, and accessible) of a web-based service and include youthful imagery and professional logos that elicit trust. In addition, we learnt that young people would want this service to be promoted through digital communications (eg, dating apps and social media), endorsement from government or other recognized institutions, and via word-of-mouth communications.

This study is one of the few studies to examine and publish findings from the design phase of developing a digital sexual health intervention and one of the only studies to publish findings on the desired language and imagery of young people as the end user [22-25]. Many studies have outlined the design process but have not clearly defined language or imagery elements of the design [26-28]. However, the summative findings from these studies reflect our findings and establish that young people want to use a web-based service but require a certain level of clarity around the confidentiality and credibility of the service—they want to trust the service [26-28].

### Social Marketing and End User Engagement

Social marketing principles informed our study design and end user engagement proved useful for exploring the needs of young people. The workshops were also able to help us reaffirm our earlier findings and assumptions important to designing a service that is attractive and useful for young people. End user engagement has been used for the development of many interventions in sexual health and exemplifies the importance of involving end users and this study is no different [29]. By examining the rationale behind young people’s preferences in a focus group setting, we are able to better tailor a youthful yet trustworthy service to young people. It is important to note that this is just the first step in the development of this service. Engaging end users in design is an iterative process and will require further involvement with young people through further user testing of prototypes created and feedback throughout implementation.

In this study, we focused on Andreasen’s principles of segmentation, exchange, and the marketing mix (product, place, price, and promotion) (Multimedia Appendix 2). Segmentation was an important principle for us to explore in this study, as our formative research suggested that some populations expressed different preferences for using web-based STI/BBV testing services [12]. While many social marketing researchers have shifted away from the use of geographic and demographic segmentation because of its failure to predict behavior change [30], we included geographic segmentation because our earlier research found that young people living in regional areas expressed a stronger preference for web-based STI/BBV testing services than metropolitan young people [12]. However, in our focus group workshops, we found overall agreement among regional and metropolitan young people on the elements of design and promotion, although regional participants placed more value on the collateral materials (eg, posters) to promote the service. This suggests that while regional youth may be more likely to want to access a web-based rather than in-person service, their preferences for the design of the service are similar to metropolitan youth.

Ideally, we would have also included segmentation on psychographic factors such as personality, social status, or opinions, but this type of segmentation is expensive and time consuming to implement in practice, given the unreliability of and complexity in collecting these data and not possible within the budgetary constraints of our project [31]. In fact, most studies do not even report on segmentation at all [32].

In our exploration of the design of the service (product) and it being web-based (place), we found that young people continuously mentioned the importance of establishing trust among potential users. Whether directly or indirectly, participants made it clear that explicit messages and imagery highlighting the confidentiality and credibility of the service were critical in establishing the level of trust needed to use the service.

Andreasen’s benchmark “exchange” (ie, motivation to engage with the service for the offer of something beneficial in return) [4] was indirectly explored throughout the study with the most interesting finding being that our traditional assumption of STI/BBV behavior being intrinsically motivated was not applicable to all participants. Our finding that some participants were more motivated by the extrinsic motivation of “protecting their partner” indicated the need to diversify messaging to extend our reach. Testing a diverse set of messages yielded important additional insights not observed in the initial survey. While the “fear-based” message (“Itching. Burning. Rash. Sores. Getting tested is a MUST!”) was one of the top selected messages in the survey, upon deeper discussion with the focus group, this was clearly found to not be a motivating message. Although not intentionally explored in this study, there were some suggestions that the messages presented could be acceptable across multiple platforms (including digital and traditional place-based advertisements). This is something we will explore further in the future.

Competition is another principle often underreported in social marketing interventions [31] and although not directly explored in this study, participants did have some important insights to inform competition. Throughout the workshops, participants made it clear that they wanted a service that had no out-of-pocket costs. This is important given our finding that there are no free web-based comprehensive STI/BBV testing services available in Victoria [10]. Those in regional areas also mentioned the potential for improved access to testing with a web-based service, as it is difficult to access the limitedly available in-person services in their geographic locations. Despite these advantages, we anticipate that strong digital promotion will be needed to compete with existing web-based services that already have high visibility.

### Limitations

Although our findings are informed by a diversity of views among young people of different genders, place of residence, and cultural backgrounds, our sample size was relatively small and did not include individuals with disabilities, those younger than 18 years, or many heterosexual men who may have different views about the messaging and imagery of a web-based STI/BBV testing service. As such, our findings may be less applicable to those groups and additional strategies may be needed to improve accessibility for these groups.

Despite the known limitations and lack of insights offered by geographic segmentation [30], we believed that in our context it was important to include this. While our focus groups were limited by lack of segmentation for other factors such as social status, lifestyle, and opinions, we will have the opportunity to explore these during phases 4 and 5 of the project—user testing and evaluation.

In this phase of developing the service, our objective was to explore imagery and messaging of a web-based service that would be acceptable to young people, rather than investigating what would make young people change their behavior and increasingly test. This will be explored during phase 5, the evaluation. We also acknowledge that while we worked directly with young people to design the imagery, messaging, and promotion of our web-based service, the clinical and legal requirements of providing web-based STI/BBV testing service in Australia required that some of the material we presented to the young people was researcher driven, rather than end user driven, potentially introducing researcher bias to the project. However, workshops did allow the young people to introduce new ideas for the design and functionality of the service and its messaging and promotion and gave important insights into young people’s intentions, motivations, and interest in web-based STI/BBV testing.

### Conclusions

This study has highlighted the value of applying social marketing theory with end user engagement when developing a web-based STI/BBV testing service. We were able to identify that young people are more likely to be attracted to a web-based service that uses messages that highlight the functional benefits of service, and the use of professional imagery and logos within the service can elicit trust. The results of this study will be used to develop and implement a web-based sexual health service for young Victorians, which will be accessible to young people and effectively increase STI/BBV testing uptake. Future research will measure the reach, the efficacy of social marketing strategy, and how this service complements existing services in increasing STI/BBV testing uptake among young Victorians.

## Appendix

Multimedia Appendix 1 Key messages, scenarios, and landing page samples used during the initial survey of young people aged 18-29 years in Victoria, Australia, exploring messaging and design preferences for a web-based sexually transmitted infection/blood-borne virus testing service.

Multimedia Appendix 2 Using Andreasen’s theory of social marketing to guide the design of a web-based sexually transmitted infection testing service for 18-29-year-olds in Victoria, Australia, with research strategies and key findings from formative research and end user engagement.

## Abbreviations

BBV: blood-borne virus
EOI: expression of interest
STI: sexually transmitted infection

## Data Availability

All data generated or analyzed during this study are available from the corresponding author on reasonable request and with appropriate ethical approval.

## References

[1] Kirby Institute, University of New South Wales (UNSW) (2022). HIV, viral hepatitis and sexually transmissible infections in Australia: annual surveillance report 2022.

[2] Turner KME, Looker KJ, Syred J, Zienkiewicz A, Baraitser P (2019). Online testing for sexually transmitted infections: A whole systems approach to predicting value. PLoS One.

[3] van Gemert-Pijnen JEWC, Nijland N, van Limburg M, Ossebaard HC, Kelders SM, Eysenbach G, Seydel ER (2011). A holistic framework to improve the uptake and impact of eHealth technologies. J Med Internet Res.

[4] Andreasen AR (2002). Marketing Social Marketing in the Social Change Marketplace. Journal of Public Policy & Marketing.

[5] Teal G, McAra M, Riddell J, Flowers P, Coia N, McDaid L (2022). Integrating and producing evidence through participatory design. CoDesign.

[6] Eysenbach G (2008). Medicine 2.0: social networking, collaboration, participation, apomediation, and openness. J Med Internet Res.

[7] Willmott TJ, Schmidtke DJ, McLeod S (2024). Editorial: Nothing about us without us: participatory design application in social marketing. JSOCM.

[8] Rundle-Thiele S, Dietrich T, Carins J (2021). CBE: A Framework to Guide the Application of Marketing to Behavior Change. Social Marketing Quarterly.

[9] Australian STI Management Guidelines for Use in Primary Care (2021). Australasian society for HIV, viral hepatitis and sexual health medicine.

[10] Cardwell ET, Ludwick T, Fairley C, Bourne C, Chang S, Hocking JS, Kong FYS (2023). Web-Based STI/HIV Testing Services Available for Access in Australia: Systematic Search and Analysis. J Med Internet Res.

[11] Ludwick T, Walsh O, Cardwell ET, Chang S, Kong FYS, Hocking JS (2024). Moving Toward Online-Based Sexually Transmitted Infection Testing and Treatment Services for Young People: Who Will Use It and What Do They Want?. Sex Transm Dis.

[12] Cardwell ET, Walsh O, Chang S, Coombe J, Fairley C, Hocking JS, Kong FYS, Ludwick T (2024). Preferences for online or in-person STI testing vary by where a person lives and their cultural background: a survey of young Australians. Sex Transm Infect.

[13] Walsh O, Cardwell ET, Hocking JS, Kong FYS, Ludwick T (2024). Where would young people using an online STI testing service want to be treated? A survey of young Australians. Sex Health.

[14] Reynolds-Tylus T (2019). Psychological Reactance and Persuasive Health Communication: A Review of the Literature. Front. Commun.

[15] Brickman J, Willoughby JF (2017). ‘You shouldn’t be making people feel bad about having sex’: exploring young adults’ perceptions of a sex-positive sexual health text message intervention. Sex Education.

[16] Jang Eunice Eunhee, Wagner Maryam, Sinclair Jeanne (2025). National Survey on Essential Communication Skills to Address Language Demands in Canadian Nursing Practice. J Adv Nurs.

[17] Denison HJ, Bromhead C, Grainger R, Dennison EM, Jutel A (2017). Barriers to sexually transmitted infection testing in New Zealand: a qualitative study. Aust N Z J Public Health.

[18] Ten Hoor GA, Ruiter RAC, van Bergen JEAM, Hoebe CJPA, Dukers-Muijrers NHTM, Kok G (2016). Predictors of Chlamydia Trachomatis testing: perceived norms, susceptibility, changes in partner status, and underestimation of own risk. BMC Public Health.

[19] Martin-Smith HA, Okpo EA, Bull ER (2018). Exploring psychosocial predictors of STI testing in University students. BMC Public Health.

[20] Corcoran N (2007). Theories and models in communicating health messages. Communicating Health: Strategies for Health Promotion.

[21] Braun V, Clarke V (2006). Using thematic analysis in psychology. Qualitative Research in Psychology.

[22] Wilkinson TA, Jenkins K, Hawryluk BA, Moore CM, Wiehe SE, Kottke MJ (2022). Dual Protection Messaging for Adolescents and Young Adults in the Setting of Over-the-Counter Hormonal Contraception: A Human-Centered Design Approach. J Pediatr Adolesc Gynecol.

[23] Njoki N, Cutherell M, Musau A, Mireri D, Nana-Sinkam A, Phillips M (2023). Applying Human-Centered Design to Replicate an Adolescent Sexual and Reproductive Health Intervention: A Case Study of Binti Shupavu in Kenya. Glob Health Sci Pract.

[24] Davis AC, Wright CJ, Murphy S, Dietze P, Temple-Smith MJ, Hellard ME, Lim MS (2020). A Digital Pornography Literacy Resource Co-Designed With Vulnerable Young People: Development of "The Gist". J Med Internet Res.

[25] Ippoliti N, Sekamana M, Baringer L, Hope R (2021). Using Human-Centered Design to Develop, Launch, and Evaluate a National Digital Health Platform to Improve Reproductive Health for Rwandan Youth. Glob Health Sci Pract.

[26] Gkatzidou V, Hone K, Sutcliffe L, Gibbs J, Sadiq ST, Szczepura A, Sonnenberg P, Estcourt C (2015). User interface design for mobile-based sexual health interventions for young people: design recommendations from a qualitative study on an online Chlamydia clinical care pathway. BMC Med Inform Decis Mak.

[27] Martin P, Alberti C, Gottot S, Bourmaud A, de La Rochebrochard E (2023). Young people's proposals for a web-based intervention for sexual health promotion: a French qualitative study. BMC Public Health.

[28] Shoveller J, Knight R, Davis W, Gilbert M, Ogilvie G (2012). Online Sexual Health Services: Examining Youth’s Perspectives. Can J Public Health.

[29] Fakoya I, Cole C, Larkin C, Punton M, Brown E, Ballonoff Suleiman A (2022). Enhancing Human-Centered Design With Youth-Led Participatory Action Research Approaches for Adolescent Sexual and Reproductive Health Programming. Health Promot Pract.

[30] Dietrich T, Hurley E, Carins J, Kassirer J, Rundle-Thiele S, Palmatier RW, Merritt R, Weaven SK, Lee N (2022). 50 years of social marketing: seeding solutions for the future. EJM.

[31] McKercher B, Tolkach D, Eka Mahadewi NM, Byomantara DGN (2022). Choosing the Optimal Segmentation Technique to Understand Tourist Behaviour. Journal of Vacation Marketing.

[32] Kubacki K, Rundle-Thiele S, Pang B, Carins J, Parkinson J, Fujihira H, Ronto R, Dietrich T, Rundle-Thiele S, Kubacki K (2017). Segmentation in social marketing: process, methods and application. An Umbrella Review of the Use of Segmentation in Social Marketing Interventions.

