# Supplementary material for: Engaging End Users to Inform the Design and Social Marketing Strategy for a Web-Based Sexually Transmitted Infection/Blood-Borne Virus (STI/BBV) Testing Service for Young People in Victoria, Australia: Qualitative Study
Source: J Med Internet Res. 2025 Mar 27;27:e63822. doi: 10.2196/63822 (PMC11986377; doi:10.2196/63822)
Supplement: Multimedia Appendix 1 [file jmir_v27i1e63822_app1.docx]

**Multimedia Appendix 1.** Key messages, scenarios and landing page samples used during the initial survey of young people 18-29 years of age in Victoria, Australia exploring messaging and design preferences for a web-based STI/BBV testing service.

| **Key Message Scenarios** | | **Key Message** |
| --- | --- | --- |
| **Scenario 1:** You went on a weekend holiday with your friends and had a great time. While on your trip, you had a few wild nights that led to a couple of hookups. There was some alcohol involved on these nights and you weren’t exactly thinking about condoms. When you get home from the holiday you are wondering if it would be a good idea to get tested for STIs. | | *Itching. Burning. Rash. Sores. Getting testing is a MUST! |
| **Scenario 2:** Your friend recently opened up to you about their sexuality. Your friend identifies as male and is attracted to other men. He told you that he has been having anonymous hookups with men from Grindr and sometimes visits the local cruising spots. He has some concerns about being ‘outed’ but wants to be tested for STIs and has asked you to help find a testing service. | | *Confidential, affordable STI testing. Find out more! |
| **Scenario 3:** You were having sex with someone for about 3 months, but things have now ended. To your knowledge, you and your partner were monogamous throughout the relationship. You are thinking it would be a good idea to get tested for STIs before you begin seeking new partners. | | *Getting tested can protect your partners from STIs. |
| **Scenario 4:** Yesterday you received a text from one of them saying, “Yo, I got tested yesterday and I have clamidia” After a brief convo with your mate, you’re still not sure what to do. | | *Be healthy. Be safe. Get tested. |
| **Scenario 5:** Last night, you had sex with your partner for the first time. You woke up this morning with some anxiety about the situation and really want to get more information about your sexual health. | | Sexual health is important. Choose to get tested today! |
| **Scenario 6:** Casual hookups are kind of your thing. You aren’t ready for anything serious but sometimes you have an itch you need to scratch. Yesterday you noticed some red spots and itching while you were peeing. This morning you noticed some unusual discharge also. | | Left untreated STIs can lead to infertility. Get tested today! |
| **Scenario 7:**You decided that you want to get a test for STIs but you have some concerns. You still live with your parents and are worried if you use your Medicare card they will find out. You don’t have the money to pay for a private service and you don’t know where to go. | | Anyone can get an STI. Get tested today! |
|  |  | Don’t go viral, unless it’s on TikTok. Get tested today! |
|  |  | You don’t have to have symptoms to have an STI. Get tested today! |
| *Indicates the most selected messages in the initial survey, which were used during the focus group workshops | | |
| **Sample landing pages** | | |
| 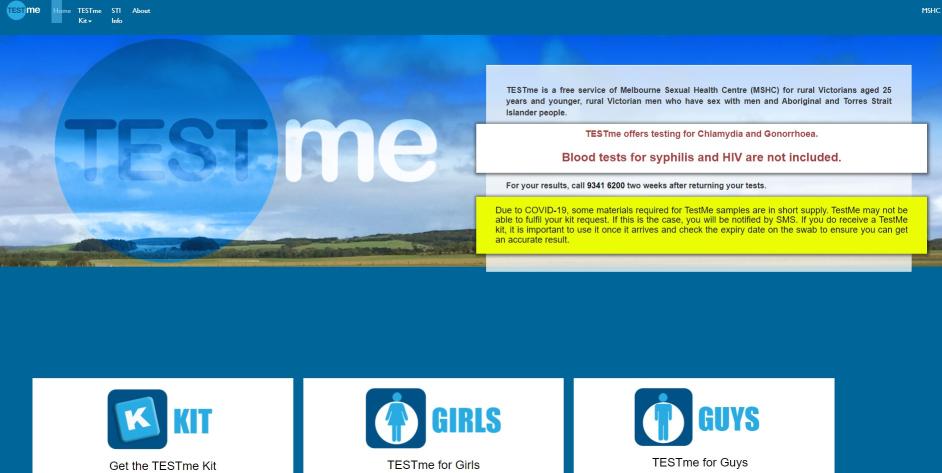 | Option 1 | |
| 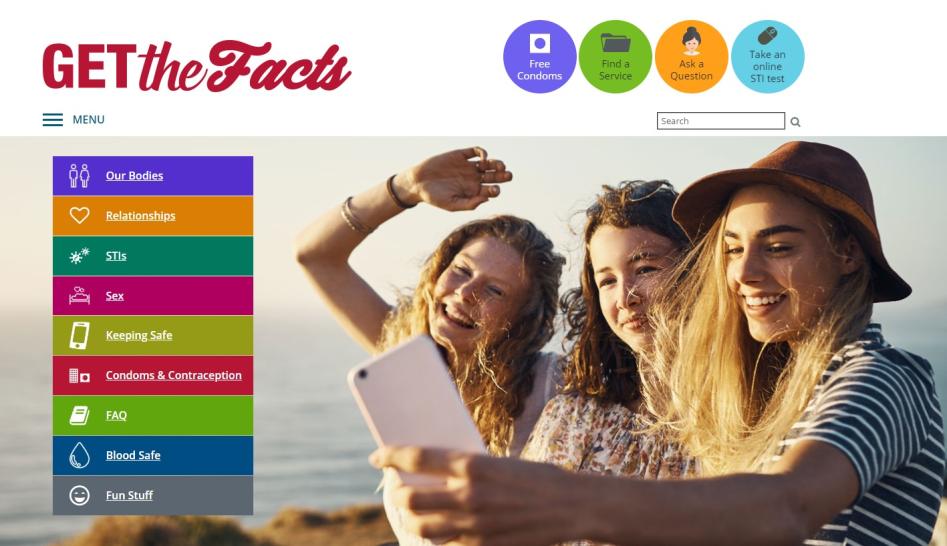 | Option 2 | |
