# Supplementary material for: Engaging End Users to Inform the Design and Social Marketing Strategy for a Web-Based Sexually Transmitted Infection/Blood-Borne Virus (STI/BBV) Testing Service for Young People in Victoria, Australia: Qualitative Study
Source: J Med Internet Res. 2025 Mar 27;27:e63822. doi: 10.2196/63822 (PMC11986377; doi:10.2196/63822)
Supplement: Multimedia Appendix 2 [file jmir_v27i1e63822_app2.docx]

**Multimedia Appendix 2.** Using Andreasen’s theory of social marketing to guide the design of a web-based STI testing service for 18–29-year-olds in Victoria, Australia with research strategies and key findings from formative research and end-user engagement.

| **Andreasen’s benchmarks** | **Definition** | **Strategy used in this study** | **Key findings** |
| --- | --- | --- | --- |
| Behavior change | measurable behavior changes the intervention seeks to address | Utilizing an iterative human centered design approach | A majority of young people engaged in the study and formative research have expressed a preference for online STI testing compared to traditional testing. There will be a comprehensive evaluation of our service once implemented that will capture impact outcomes such as use of the service, impact on testing uptake, and implementation outcomes such as acceptability, adoption, and sustainability. |
| Formative research | understanding of user experiences, values and needs | Surveyed 900 young Australians and reviewed literature and existing online STI testing services to gather important insights | Online STI testing and online service delivery varied among demographic population, particularly among those living in geographically regional populations (11-13) |
| Segmentation of target audience | aggregation of users into subgroups based on things like demographics, behavior, or geographic location | Recruited participants and allocated them to focus group workshops based on differing preferences/needs according to formative research | Based on formative research, we found that young Australian’s preferences for online STI testing and online service delivery varied among demographic population, particularly among those living in geographically regional populations (11-13). This research encouraged the decision to segment based on geographic location. However, key findings from this study indicate consensus in preferences across all geographic locations. |
| Exchange | what will motivate users to engage with the service for the offer of something beneficial in return | Used focus group workshops to explore factors that motivate young people to use an online STI/BBV and their perceived benefits of the service | The main motivating factors for using an online STI testing service highlighted by the participants in this study are privacy and access. Participants frequently made comments on the benefit of increased privacy and ability to access a testing service online and how these were motivating factors. They also made comments on how their motivation for testing could be intrinsic or altruistic depending on the person. |
| Marketing mix | the 4Ps of traditional marketing; product, price, place and promotion | Used focus group workshops to investigate:  Product in how messaging and imagery for this service could best be tailored to young people as the end user  Place in how we explore the positive and negative aspects of an online testing service  Price was explored with willingness to pay for the service  Promotion in the discussion of how young people find health services | Product: participants gave important insights on messaging and imagery. They preferred language with a comforting, non-judgement tone and messaging that focused on the functional benefits of the service (i.e. confidentiality, affordability, accessible online). They preferred imagery that would strike a balance between traditional online medical imagery that they recognized and trusted and the youthful imagery they were most attracted to.  Place: participants frequently mentioned privacy as a positive aspect of this service. However, this aspect was leveraged on the service’s ability to establish itself as trustworthy. One aspect mentioned by some participants was not being able to consult further with a clinician, as you would with traditional in-person services.  Price: participants made it clear that the service should be provided at no out-of-pocket cost.  Promotion: participants mentioned several avenues as to how the service could be promoted to them; social media, word-of-mouth (trusted friends and healthcare providers), search engine optimization/promotion and collateral material in frequented public locations |
| Competition | competing behaviors and strategies to reduce the competition | Explored indirectly with insights from the survey, review of literature and existing services, and focus groups workshops | Although this is a supplemental service, in both this study and formative research participants made comments that elude to the superiority of our service as it offers easily accessible, comprehensive STI/BBV testing at no out-of-pocket cost |
